# A small supernumerary marker chromosome present in a Turner syndrome patient not derived from X- or Y-chromosome: a case report

**DOI:** 10.1186/1755-8166-2-22

**Published:** 2009-11-12

**Authors:** Frenny Sheth, Elisabeth Ewers, Nadezda Kosyakova, Anja Weise, Jayesh Sheth, Manisha Desai, Joris Andrieux, Joris Vermeesch, Ahmed B Hamid, Monika Ziegler, Thomas Liehr

**Affiliations:** 1 Institute of Human Genetics, Foundation for Research In Genetics and Endocrinology [FRIGE], Jodhpur Gam Road, Satellite, Ahmedabad-380 015., India; 2 Jena University Hospital, Institute of Human Genetics and Anthropology, Kollegiengasse 10, D-07743 Jena, Germany; 3 Laboratory of Medical Genetics, Jeanne de Flandre Hospital CHRU de Lille, Lille Cedex, France; 4 Center for Human Genetics, K.U.Leuven, Herestraat 49, 3000 Leuven, Belgium

## Abstract

**Background:**

Small supernumerary marker chromosomes (sSMC) can be present in numerically abnormal karyotypes like in a 'Turner-syndrome karyotype' mos 45,X/46,X,+mar.

**Results:**

Here we report the first case of an sSMC found in Turner syndrome karyotypes (sSMC^T^) derived from chromosome 14 in a Turner syndrome patient. According to cytogenetic and molecular cytogenetic characterization the karyotype was 46,X,+del(14)(q11.1). The present case is the third Turner syndrome case with an sSMC^T ^not derived from the X- or the Y-chromosome.

**Conclusion:**

More comprehensive characterization of such sSMC^T ^might identify them to be more frequent than only ~0.6% in Turner syndrome cases according to available data.

## Background

Small supernumerary marker chromosomes (sSMC) [1] can be observed in a numerically normal 'basic karyotype', but also in numerically abnormal one like in a 'Turner-syndrome karyotype' (=sSMC^T^). At present 528 such cases with an sSMC^T ^are reported [2,3]. sSMC^T ^are very rare in the common population (1:100000 [2]) - however, they can be observed 45 and even 60 times more frequent in infertile and developmentally and/or mentally retarded patients, respectively. The majority of sSMC^T^(X) form ring-chromosomes, while most sSMC^T^(Y) are inverted duplicated/isodicentric ones. When a mos 45,X/46,X,der(Y) or 45,X/46,XY is characterized it is important to counsel the patient concerning a possibility of gonadoblastoma and a preventive removal of gonadal tissue. In this connection, the necessity to apply molecular approaches for detection of cryptic 45,X/46,XY mosaicism is discussed, as a direct relationship between percentage of cells exhibiting a 45,X karyotype and patients phenotype does not exist. Additionally, it is a well-known fact that in a karyotype of mos 45,X/46,X,der(X) it is important to test for the ability of the der(X) to be inactivated, i.e. to test for the presence of the XIST-gene [2].

Even though sSMC^T ^derive in >99% of the cases from one of the gonosomes, there are also two previous exceptional reports on sSMC^T ^derived from one of the autosomes [4,5].

Here we report the third case with an sSMC^T ^originating not from a gonosome but the first one proven to be derived from chromosome 14.

## Case presentation

A ten year old girl was studied cytogenetically due to typical features of a Turner syndrome, i.e. short stature, webbing of neck, cubitus valgus, shield chest, congenital dislocation of hip, renal anomalies, clinodactyly, unilateral simian crease on right palm, acyanotic congenital heart disease and small patent ductus arteriosus.

## Results

**Figure 1:**
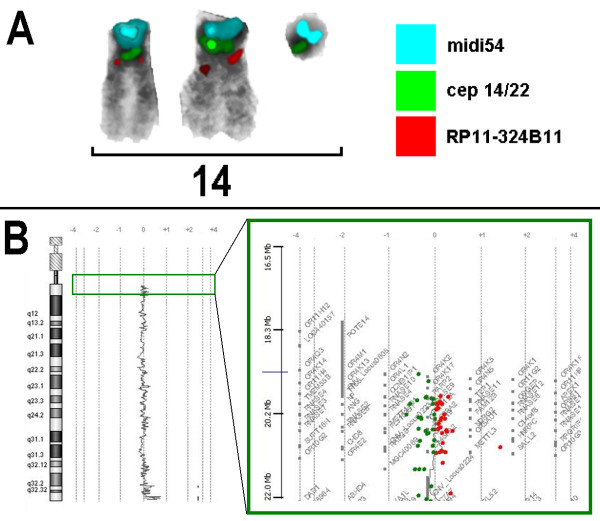
**A) SubcenM-FISH revealed the absence of euchromatic material on the sSMC^T ^reported here**. The sSMC^T ^only showed one specific signal, each, for midi 54 (a probe specific for the acrocentric short arms) and the centromeric probe specific for chromosome 14 and 22 (cep 14/22). No specific signals were on the sSMC^T ^for the centromere-near probe RP11-324B11 in 14q11.2 and partial chromosome painting probe of chromosome 14 (the latter not depicted here). Thus, the sSMC^T ^was a del(14)(q11.1). B) After knowing the origin of the sSMC array-CGH was reanalyzed for 14q-proximal region. The first probe on Agilent 4x44K array location is on 19,365,051-19,365,110 at 14q11.2. Thus, only genes from olfactory receptors OR11H12, OR4M1 and OR4Q3, and POTEG and P704P may be involved in the marker chromosome. As it is a region known to be CNV polymorphic and the probe RP11-324B11 at 19,886,099-19,886,646 is not present on the marker according to FISH array-CGH overall is to be considered as non-informative for this sSMC.

Cytogenetics revealed a karyotype 46,X,+mar in a patient with Turner syndrome. The sSMC^T ^was acquired de novo, as parental chromosome analysis revealed. Array-CGH was done, however no clear imbalance apart from the lack of a second gonosome was observed (see also Fig. 1B). cenM-FISH identified the sSMC^T ^as a derivative of chromosome 14. In cenM-FISH the sSMC^T ^did only show a signal for the probe D14/22Z1, but not for D22Z4 specific for the centromeric region of chromosome 22; thus, the sSMC^T ^could be defined as a der(14). By subcenM-FISH and the array-CGH result was confirmed that the sSMC^T ^did not contain euchromatic material and it could be defined as a del(14)(q11.1) (Fig. 1A).

## Discussion

Here we report the third case of a patient with sSMC^T ^not derived from a gonosome. It is the first such case where the sSMC was characterized in detail by molecular cytogenetics and which turned out to be a de novo derivative of chromosome 14. Previously one case with a der(20) [4] and a not further specified sSMC^T^, however, proven to be not of gonosomal origin [5] were characterized. Overall, this is an interesting finding as neither chromosome 15 nor 22 were up to now identified as sSMC^T^, even though these two chromosomes are most frequently involved in sSMC formation [1,3]. However, this might only be a bias due to only three known cases up to now. Furthermore, the exclusion of a uniparental disomy 14 would have been desirable; unfortunately no paternal material was available for that kind of study.

Among ~3.400 reported sSMC cases studied for their chromosomal origin and subsequently reported [3], by now 528 cases with sSMC^T ^were found. Three of those sSMC^T ^were not of gonosomal origin, i.e. 0.6%. However, the question is, if the percentage of this specific kind of sSMC^T ^is not underestimated. Non-gonosomal sSMC^T ^might be easily missed if they are not further characterized by molecular approaches.

In conclusion, a really comprehensive characterization of all sSMC by different probes, probe sets and approaches could enhance the detection rate of autosomal derived sSMC^T^.

## Materials and methods

### Cytogenetics

Metaphase chromosome preparations were obtained from PHA stimulated lymphocyte cultures according to standard procedures. Chromosome analysis was carried out applying GTG banding at a 600 band level according ISCN 2009 [6] in the patient (25 metaphases) and both parents (50 metaphases, each).

### Fluorescence in situ hybridization (FISH)

FISH was performed as previously reported [7]. To characterize the sSMC first centromere specific multicolor FISH (cenM-FISH) and then subcentromere-specific M-FISH (subcenM-FISH) was performed; for details see [7]. The here applied probe RP11-324B11 in 14q11.2 is located at 19,886,099-19,886,646 Mb.

### Array-CGH

Genomic DNA was extracted from peripheral blood lymphocytes using standard SDS-proteinase K extraction method [8]. DNA concentration was determined with NanoDrop ND-1000 spectrophotometer and software (NanoDrop Technologies, Berlin, Germany). Detection of gene copy number was performed by array-Comparative Genomic Hybridization (array-CGH) experiments following standard and manufacturer's recommendations using 44.000 oligo probes approximately spaced at 40-100 kb intervals across the genome (Human Genome CGH microarray 44B kit, Agilent™). Male genomic DNA (Promega™) was used as reference in sex-match hybridizations which were analyzed with the CGH-analytics software v3.4 by applying Z-score segmentation algorithm with a window size of 10 points to identify chromosome aberrations. Analysis was performed with filter settings: 3-point filter and 0.2 of variation.

## Consent section

Written informed consent was obtained from the patient for publication of this case report and accompanying images. A copy of the written consent is available for review by the Editor-in-Chief of this journal.

## Competing interests

The authors declare that they have no competing interests.

## Authors' contributions

FS, JS and MD performed the cytogenetic studies in the present case and collected the data relative to this case report. EE, NK, AW, ABH, MZ and TL did the molecular cytogenetic analysis and interpretations. JA and JV were involved in the array-CGH analysis. TL drafted the paper and all authors contributed to the finalizing of the manuscript.
